# Characterizing the rhythmic oscillations of gut bacterial and fungal communities and their rhythmic interactions in male cynomolgus monkeys

**DOI:** 10.1128/spectrum.00722-24

**Published:** 2024-09-25

**Authors:** Yunpeng Yang, Meiling Yu, Yong Lu, Changshan Gao, Ruxue Sun, Wanying Zhang, Yanhong Nie, Xinyan Bian, Zongping Liu, Qiang Sun

**Affiliations:** 1 Jiangsu Co-innovation Center for Prevention and Control of Important Animal Infectious Diseases and Zoonoses, College of Veterinary Medicine, Yangzhou University, Yangzhou, China; 2 Institute of Comparative Medicine, Yangzhou University, Yangzhou, China; 3 Institute of Neuroscience, CAS Key Laboratory of Primate Neurobiology, State Key Laboratory of Neuroscience, CAS Center for Excellence in Brain Science and Intelligence Technology, Chinese Academy of Sciences, Shanghai, China; 4 Shanghai Center for Brain Science and Brain-Inspired Technology, Shanghai, China; Central Texas Veterans Health Care System, Temple, Texas, USA

**Keywords:** gut microbiota, diurnal oscillation, bacterial and fungal microbes, male cynomolgus monkeys, correlations

## Abstract

The circadian oscillation of gut microbiota plays vital roles in the normal physiology and health of the host. Although the diurnal oscillation of intestinal bacteria has been extensively studied, little relevant work has been done on intestinal fungi. Besides, the rhythmic correlations between bacterial and fungal microbes are also scarcely reported. Here, we investigated the diurnal oscillations of bacterial and fungal communities in male cynomolgus monkeys by performing 16S rRNA and ITS amplicon sequencing. As for bacterial genera, we found that the relative abundance of *Prevotella*, norank_f_Eubacterium_coprostanoligenes_group, and *Peptococcus* underwent significant changes at ZT12 (19:00) and exhibited obvious rhythmic oscillations. Consequently, most of the bacterial functions varied at ZT12 and were positively correlated with the bacterial genera norank_f_Eubacterium_coprostanoligenes_group and *Prevotella*. Among the fungal genera, the relative abundance of *Aspergillus* and *Talaromyces* decreased at ZT18 (1:00) and showed slight rhythmic oscillations. As for the fungal function, the undefined saprotroph showed slight rhythmic oscillation and was positively correlated with the fungal genus *Aspergillus*. Notably, we characterized the correlations between intestinal bacteria and fungi every 6 h over the course of a day and found that the bacterial and fungal microbes interacted closely, with the most bacteria–fungi interactions occurring at ZT12. Our study contributed to a more comprehensive understanding of the diurnal oscillation patterns of bacterial and fungal microbes in male cynomolgus monkeys and uncovered their correlations during a diurnal cycle.

**IMPORTANCE:**

The rhythmic oscillation of gut microbiota can impact the physiology activity and disease susceptibility of the host. Until now, most of the studies are focused on bacterial microbes, ignoring other components of gut microbes, such as fungal microbes (mycobiota). Besides, only few studies have addressed the rhythmic correlations between gut bacteria and fungi. Here, we analyzed the rhythmic oscillations of bacterial and fungal communities in male cynomolgus monkeys by performing 16S rRNA and ITS amplicon sequencing. Apart from identifying the rhythmically oscillated bacterial and fungal microbes, we conducted the correlation analysis between these two microbial communities and found that the intestinal bacteria and fungi exhibited close interactions rhythmically, with the most interactions occurring at ZT12. Thus, our study not only investigated the rhythmic oscillations of gut bacterial and fungal communities in male cynomolgus monkeys but also uncovered their rhythmic interactions.

## INTRODUCTION

The circadian clock system, which consists of several transcription factors, is a complex and highly specialized network (1). It synchronizes most of the physiological activities of a host to geophysical time by driving the expression of functional genes rhythmically (2, 3). As reported, the disruption of circadian rhythm could bring various diseases, such as sleep and psychiatric disorders (4, 5), ischemic disease (6), cardiovascular dysfunction (7, 8), cancer (9), and neurodegenerative diseases (10), suggesting that circadian rhythm played vital roles in the health of the host.

Gut microbiota is a complex microbial ecosystem containing bacteria, viruses, fungi, yeasts, and other single-celled organisms. It resides in the intestinal tract of the host and exerts profound influences on their health (11, 12). Accumulating evidence indicated that the composition of gut microbiota could be affected by various factors, such as sex (13, 14), diet (15), aging (16), seasonal factor (17), delivery mode (natural birth or cesarean section) (18), gut biogeography (19), and host genetics (20, 21). Apart from these factors, the biogeography, composition, function, and metabolome of gut microbiota could also be affected by the circadian clock system and exhibit rhythmic oscillations during the 24-h light–dark cycle (22, 23). Inversely, the gut microbiota could also regulate the sleep/wake cycles of the host through gut–brain–microbiota interaction (24). Besides, gut microbial oscillation would program the circadian epigenetic and transcriptional landscape of the host and impact its physiology and disease susceptibility (25). Thus, it is of great significance to investigate the rhythmic oscillation pattern of gut microbiota.

Among the gut microbiota, most of the studies had been focused on the bacterial and fungal microbes, two important microbial communities that are closely related to the health of the host. As reported, the bacterial microbes were closely related to gastric disease (26), cardiovascular disease (27), liver disease (28), immune disease (29), and neurodegenerative disease (30, 31), while the dysbiosis of fungal microbes led to alcoholic liver disease (32), allergic airway disease (33), and gut colitis (34, 35). Although both the bacterial and fungal microbes were important for the health of the host, the researchers had focused mostly on deciphering the rhythmic oscillation mode of intestinal bacteria, while little relevant work had been done on intestinal fungi.

Non-human primates (NHPs) share similar genetic, physiological, and behavioral traits with human beings and are viewed as excellent models for scientific research and clinical application. Although the gut bacterial and fungal composition of NHPs has been extensively investigated (16, 19, 36–38), the diurnal oscillations of intestinal bacteria and fungi and their rhythmic correlations have not been reported in NHPs. In this work, we analyzed the rhythmic oscillations of bacterial and fungal communities in male cynomolgus monkeys by performing 16S rRNA and ITS amplicon sequencing and investigated the correlations between gut microbiota and the predicted microbial functions. Notably, the rhythmic interactions between bacterial and fungal microbes were uncovered by performing bacterial–fungal correlation analysis during a diurnal cycle.

## RESULTS

### Characterizing the rhythmic oscillations and correlations of bacterial microbes

**Fig 1:**
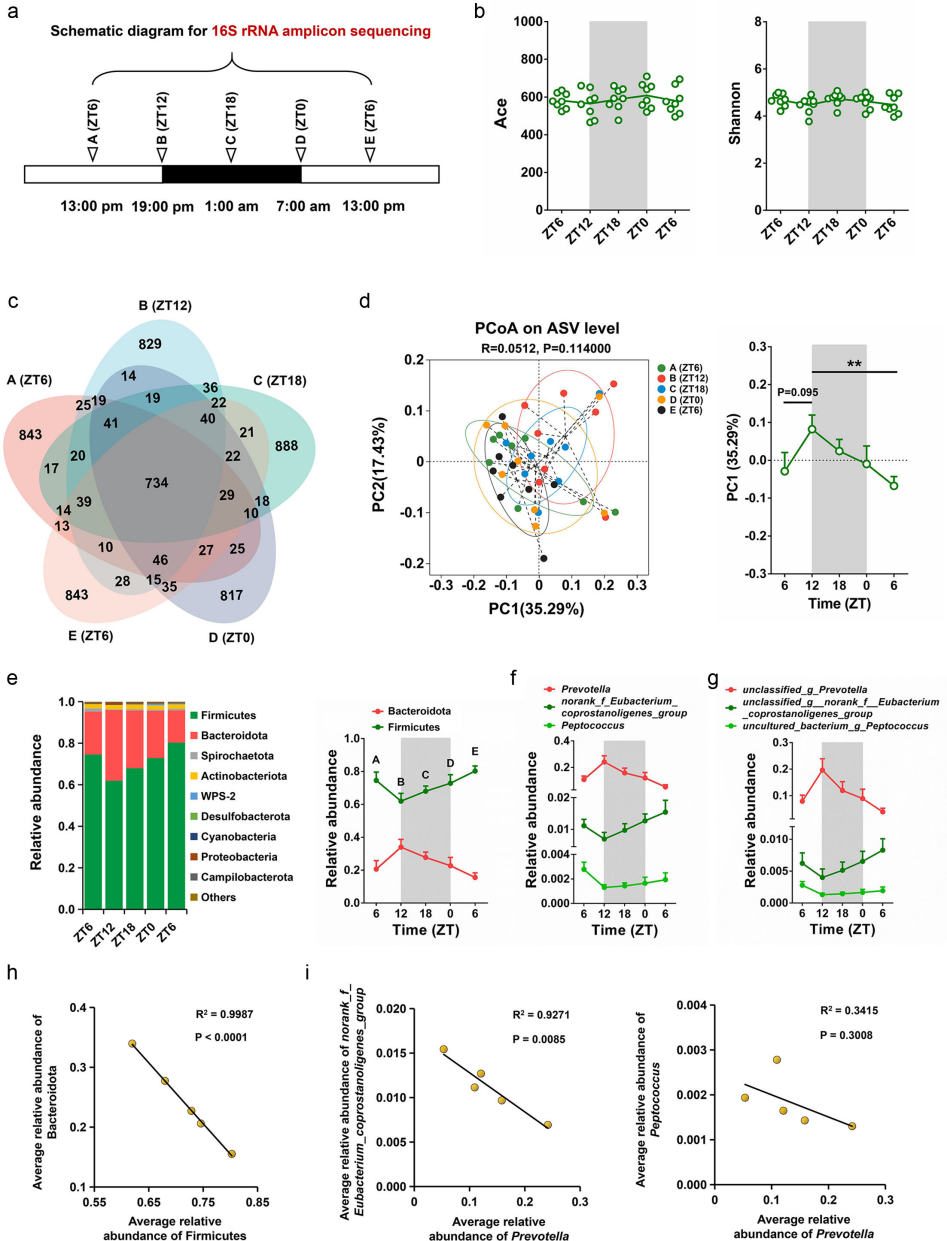
The rhythmic oscillations and interactions of bacterial microbes. (a) Schematic diagram showing the collection of fecal samples for 16S rRNA gene sequencing. The feces are collected from eight male cynomolgus monkeys with ages ranging from 2 to 3 years over zeitgeber time. (b) The circadian oscillation of Ace and Shannon index. (c) Venn diagram illustrating the number of different ASVs between the 16S rRNA gene sequencing data assayed at ZT6 (**A**), ZT12 (**B**), ZT18 (**C**), ZT0 (**D**), and ZT6 (**E**). (d) PCoA of weighted UniFrac distances based on the ASVs of five time points. The PC1 position is listed and compared between the five time points. The data are presented as the mean ± SEM. The statistical significance between ZT12 and the other time points is analyzed using a *t*-test (^*^P < 0.05; ^***^P < 0.001). (e) The relative abundance of Firmicutes and Bacteroidota over zeitgeber time. (f) The relative abundance of *Prevotella*, norank_f_Eubacterium_coprostanoligenes_group, and *Peptococcus* over zeitgeber time. (g) The relative abundance of unclassified_g_*Prevotella*, uncultured_bacterium_g_*Peptococcus*, and unclassified_g_norank_f_Eubacterium_coprostanoligenes_group over zeitgeber time. (h) Linear regression analysis for the correlation between the average relative abundance of Firmicutes and Bacteroidota. (i) Linear regression analysis for the correlations between the average relative abundance of *Prevotella* and norank_f_Eubacterium_coprostanoligenes_group and *Peptococcus*.

In this work, eight male cynomolgus monkeys with ages ranging from 2 to 3 years were selected and used to collect feces every 6 h during a 24-h period (five time points) (Fig. 1a). The measurement of routine blood indexes indicated that all these monkeys were in good health (Fig. S1). Finally, 40 fecal samples were collected and used for 16S rRNA gene sequencing (Fig. 1a). To minimize the effects of sequencing depth on α- and β-diversity measure, the number of 16S rRNA amplicon sequencing from each sample was rarefied to 67,279, which yielded an average Good’s coverage above 99% (Fig. S2A). As for microbial richness and diversity, the Ace and Shannon index showed no obvious rhythmicity during a diurnal cycle (Fig. 1b). A Venn diagram showed that 734 amplicon sequence variants (ASVs) were shared by the 16S rRNA amplicon sequencing data of five time points, while 843, 829, 888, 817, and 843 ASVs exclusively belonged to ZT6, ZT12, ZT18, ZT0, and ZT6, respectively (Fig. 1c). Principal coordinate analysis (PCoA) of weighted UniFrac distances showed a significant difference between the bacterial microbes of ZT12 and the other time points, with the principal component 1 (PC1) axis explaining a substantial proportion of the variability (Fig. 1d).

Linear discriminant analysis (LDA) effect size (LEfSe) was performed to identify the distinguishing bacterial taxa between ZT6 (A), ZT12 (B), ZT18 (C), ZT0 (D), and ZT6 (E) (LDA score >2.0). As shown in Fig. S3, *Alistipes* and uncultured_bacterium_g_*Solobacterium* were enriched at ZT6 (A), *Prevotella* and *Fournierella* were abundant at ZT12 (B), *Prevotellaceae_bacterium_DJF_LS10* and *Enterococcus* were enriched at ZT18 (C), Rhodocyclaceae was abundant at ZT0 (D), and Christensenellaceae and unclassified_g_*Monoglobus* were enriched at ZT6 (E).

To further explore the oscillation patterns of bacterial microbes, the relative abundance of microbiota at the phylum, genus, and species levels were analyzed. At the phylum level, Firmicutes and Bacteroidota showed obvious rhythmic oscillations (Fig. 1e). Specifically, the relative abundance of Firmicutes decreased at ZT12, while the relative abundance of Bacteroidota increased at the same time point. At the genus level, the genera *Prevotella*, norank_f_Eubacterium_coprostanoligenes_group, and *Peptococcus* showed obvious rhythmic oscillations (Fig. 1f). Correspondingly, the bacterial microbes unclassified_g*_Prevotella*, unclassified_g_norank_f_Eubacterium_coprostanoligenes_group, and uncultured_bacterium_g_*Peptococcus* exhibited similar rhythmic oscillations (Fig. 1g). By performing the bacteria–bacteria correlation analysis, we found that the average relative abundance of Firmicutes was negatively correlated with Bacteroidota (Fig. 1h). At the genus level, the average relative abundance of *Prevotella* was negatively correlated with the genera norank_f_Eubacterium_coprostanoligenes_group and *Peptococcus* (Fig. 1i).

Based on these results, we concluded that most of the bacterial microbes of male cynomolgus monkeys exhibited rhythmic oscillations and were closely correlated with each other during a diurnal cycle.

### The predicted gut bacterial functions and their correlations with bacterial genera

**Fig 2:**
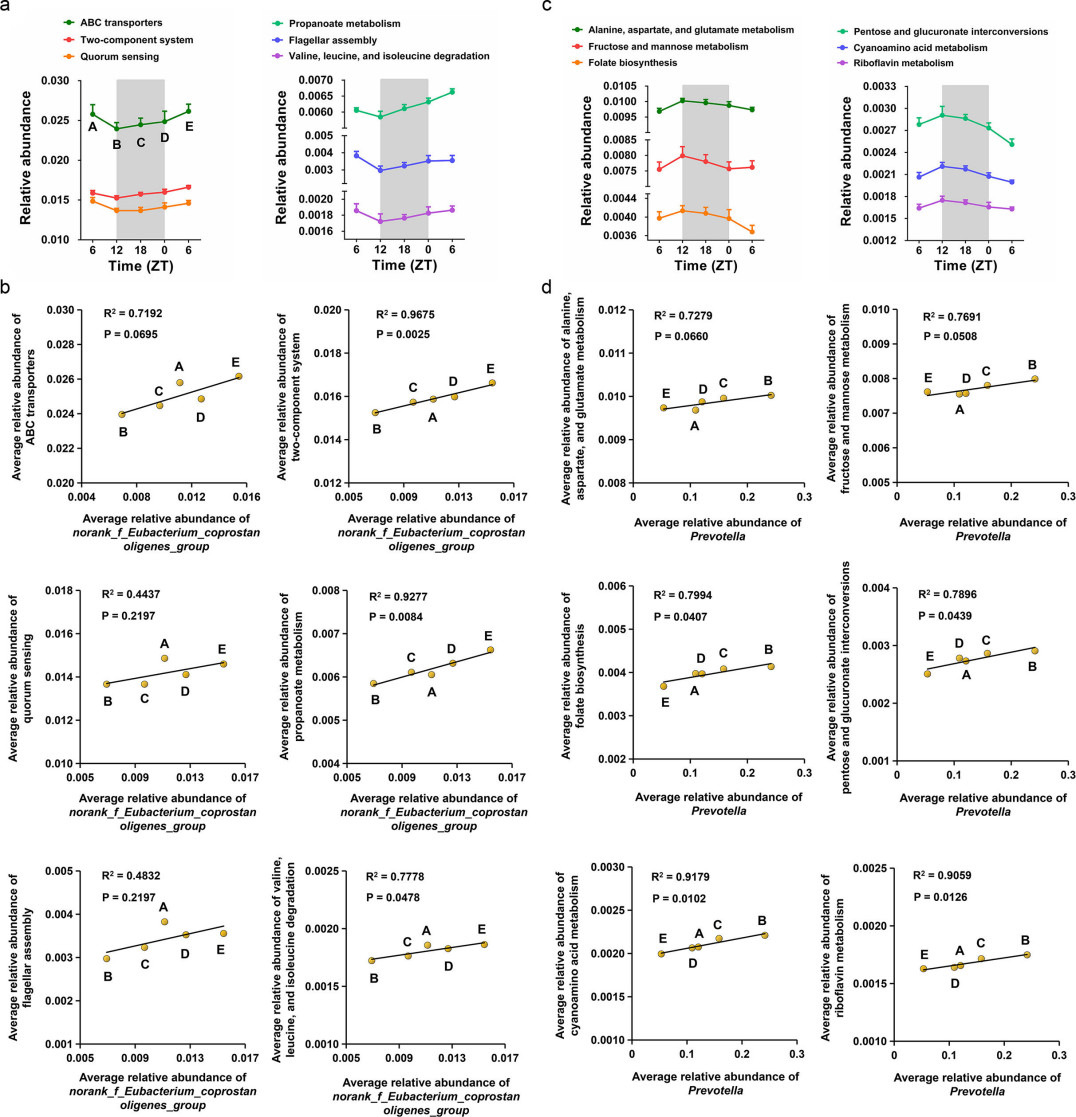
The rhythmic oscillations of gut bacterial functions and their correlations with bacterial genera. (a) The rhythmic oscillations of six gut bacterial functions whose relative abundance was decreased at ZT12. (b) Linear regression analysis for the correlations between the average relative abundance of norank_f_Eubacterium_coprostanoligenes_group and the average relative abundance of six gut bacterial functions listed in (a). (c) The rhythmic oscillations of six gut bacterial functions whose relative abundance was increased at ZT12. (d) Linear regression analysis for the correlations between the average relative abundance of *Prevotella* and the average relative abundance of six gut bacterial functions listed in (c).

Phylogenetic Investigation of Communities by Reconstruction of Unobserved States (PICRUSt2) was used for the function analysis of bacterial communities at ZT6 (A), ZT12 (B), ZT18 (C), ZT0 (D), and ZT6 (E) (Fig. S4). By focusing on the bacterial functions that showed obvious rhythmicity, we found that the relative abundance of ABC transporters, two-component system, quorum sensing, propanoate metabolism, flagellar assembly, and valine, leucine, and isoleucine degradation were decreased at ZT12 and positively correlated with the relative abundance of norank_f_Eubacterium_coprostanoligenes_group during a diurnal cycle (Fig. 2a and b). Differently, the relative abundance of alanine, aspartate, and glutamate metabolism, fructose and mannose metabolism, folate biosynthesis, pentose and glucuronate interconversions, cyanoamino acid metabolism, and riboflavin metabolism were increased at ZT12 and positively correlated with the relative abundance of *Prevotella* during a diurnal cycle (Fig. 2c and d).

### Characterizing the rhythmic oscillations of fungal microbes

**Fig 3:**
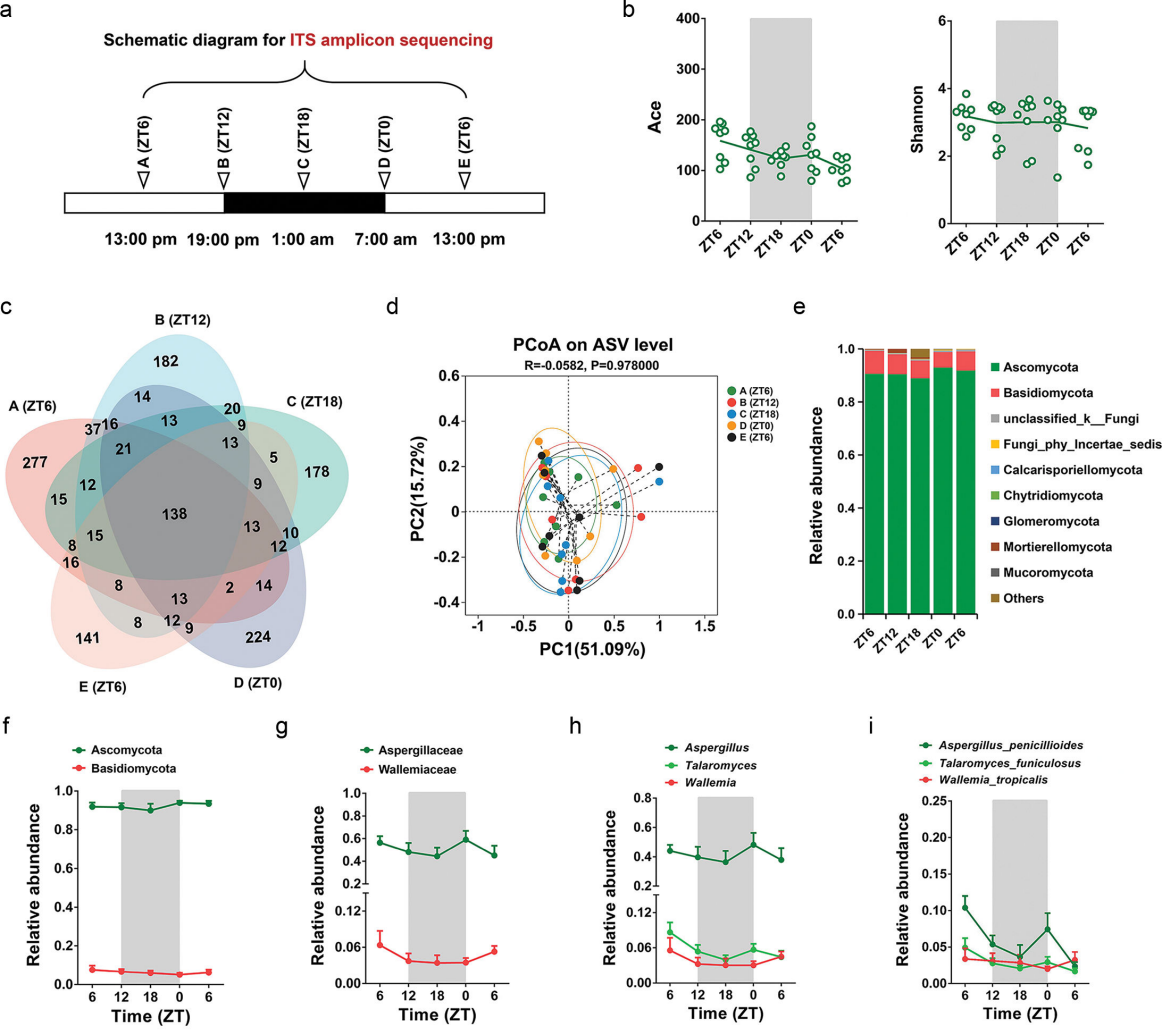
The rhythmic oscillations of fungal microbes. (a) Schematic diagram showing the collection of fecal samples for ITS amplicon sequencing. (b) The circadian oscillation of Ace and Shannon index. (c) Venn diagram illustrating the number of different ASVs between the ITS amplicon sequencing data assayed at ZT6, ZT12, ZT18, ZT0, and ZT6. (d) PCoA of weighted UniFrac distances based on the ASVs of five time points. (e) The relative abundance of top 9 phyla over zeitgeber time. (f) The relative abundance of Ascomycota and Basidiomycota over zeitgeber time. (g) The relative abundance of Aspergillaceae and Wallemiaceae over zeitgeber time. (h) The relative abundance of *Aspergillus*, *Talaromyces*, and *Wallemia* over zeitgeber time. (i) The relative abundance of *Aspergillus penicillioides*, *Talaromyces funiculosus*, and *Wallemia tropicalis* over zeitgeber time.

Based on the 40 fecal samples rhythmically collected from the eight male cynomolgus monkeys, here, we performed ITS amplicon sequencing to investigate the rhythmic oscillations of intestine fungal microbes (Fig. 3a). To minimize the effects of sequencing depth on α- and β-diversity measures, the number of ITS amplicon sequencing from each sample was rarefied to 26,513, which still yielded an average Good’s coverage above 99% (Fig. S2B). As to the microbial richness and diversity, the Ace and Shannon index showed no obvious rhythmicity (Fig. 3b). The Venn diagram showed that 138 ASVs were shared by the ITS amplicon sequencing data of five time points, while 277, 182, 178, 224, and 141 ASVs exclusively belonged to ZT6, ZT12, ZT18, ZT0, and ZT6 (Fig. 3c). PCoA of weighted UniFrac distances showed no obvious difference between the fungal microbes of five time points (Fig. 3d).

LEfSe was performed to identify the distinguishing fungal taxa between ZT6 (A), ZT12 (B), ZT18 (C), ZT0 (D), and ZT6 (E) (LDA score >1.0). As shown in Fig. S5, *Aspergillus penicillioides*, *Oidiodendron cereale*, *Talaromyces islandicus*, and *Penicillium capsulatum* were enriched at ZT6 (A); Piskurozymaceae, *Solicoccozyma*, and *Phaeoacremonium* were abundant at ZT12 (B); Herpotrichiellaceae was enriched at ZT18 (C); and *Acremonium sordidulum* and *Exophiala* were abundant at ZT6 (E). Of note, no fungal taxa abundant at ZT0 (D) was detected.

Next, the relative abundance of fungal microbes was analyzed to investigate their oscillation patterns. As shown in Fig. 3e through i, the relative abundance of Ascomycota, Aspergillaceae, *Aspergillus*, *Talaromyces*, *Aspergillus penicillioides*, and *Talaromyces funiculosus* was decreased at ZT18 and exhibited slight rhythmic oscillations.

### The predicted gut fungal functions and their correlations with fungal genera

**Fig 4:**
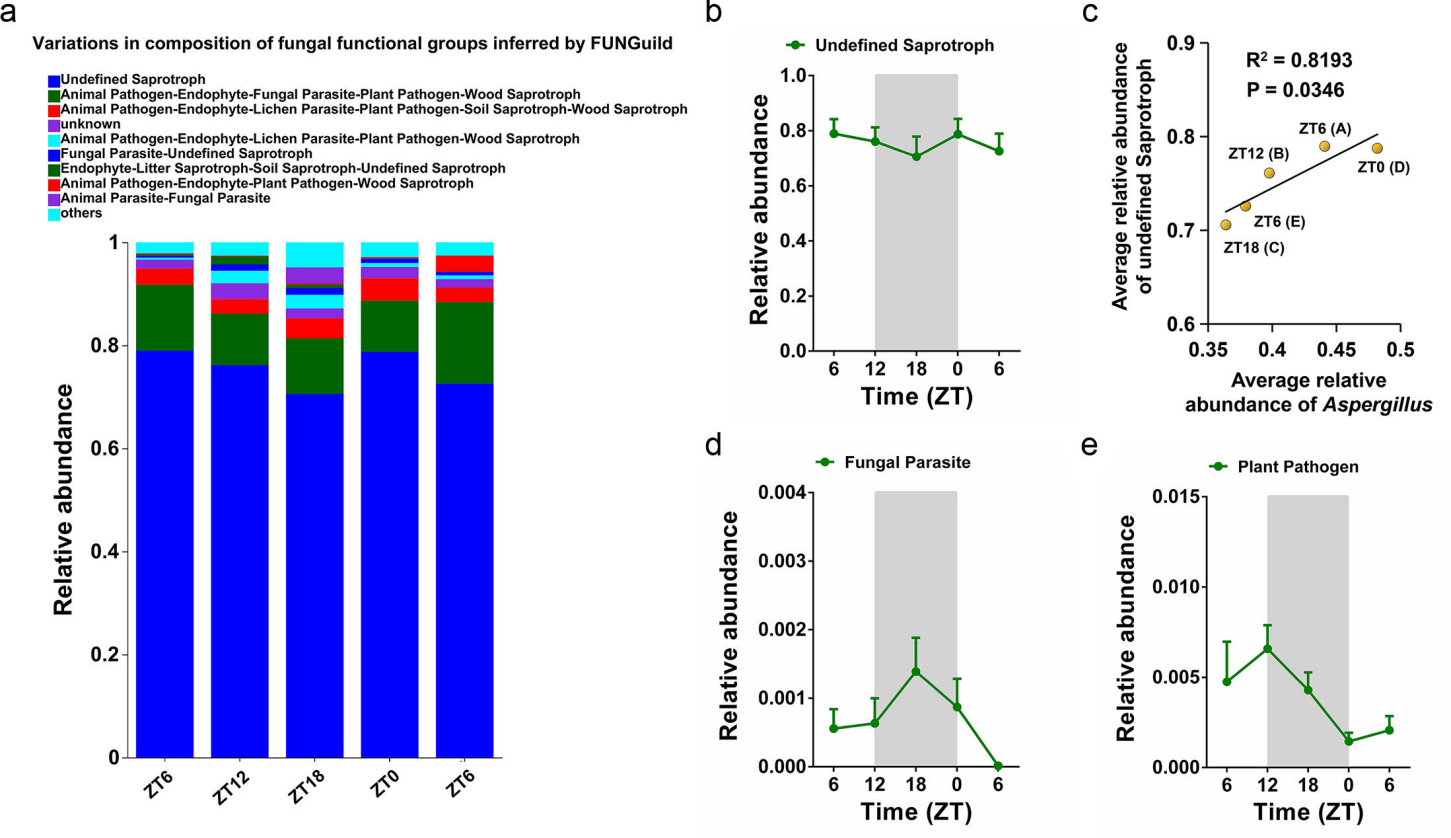
The rhythmic oscillations of gut fungal functions and their correlations with fungal genera. (a) The relative abundance of top 9 fungal functions. (b) The relative abundance of undefined saprotroph at ZT6 (**A**), ZT12 (**B**), ZT18 (**C**), ZT0 (**D**), and ZT6 (**E**). (c) Linear regression analysis for the correlation between the average relative abundance of *Aspergillus* and the average relative abundance of undefined saprotroph. (d) The relative abundance of fungal parasite at ZT6 (**A**), ZT12 (**B**), ZT18 (**C**), ZT0 (**D**), and ZT6 (**E**). (e) The relative abundance of plant pathogen at ZT6 (**A**), ZT12 (**B**), ZT18 (**C**), ZT0 (**D**), and ZT6 (**E**).

FUNGuild was used for the function prediction of fungal communities at ZT6 (A), ZT12 (B), ZT18 (C), ZT0 (D), and ZT6 (E). By focusing on the top 9 fungal functions, we found that the relative abundance of undefined saprotroph decreased at ZT18 (C) (Fig. 4a and b) and showed a similar oscillation mode with the fungal genus *Aspergillus* (Fig. 3h). By performing the correlation analysis between the fungal genera and fungal function, we found that the relative abundance of *Aspergillus* was positively correlated with the relative abundance of undefined saprotroph (Fig. 4c). Apart from undefined saprotroph, we also found that the relative abundance of fungal parasite and plant pathogen exhibited different oscillation patterns and showed no obvious correlation with the fungal genera (Fig. 4d and e).

### The correlations between bacterial and fungal genera during a diurnal cycle

We performed Spearman correlation analysis to uncover the bacterial–fungal correlations during a diurnal cycle (Tables S1 to S5). As shown in Fig. S6, 11 fungal genera (unclassified_f_Bionectriaceae, *Chaetomium*, *Bionectria*, *Botryotrichum*, *Kiflimonium*, *Acaulium*, *Chloridium*, *Clonostachys*, *Solicoccozyme*, *Cephalotrichum*, and *Pseudombrophila*) formed a positive and high-magnitude correlation network at the A (ZT6) time point. Besides, among the five time points, the bacterial and fungal microbes formed the most complicated interactions at the B (ZT12) time point.

**Fig 5:**
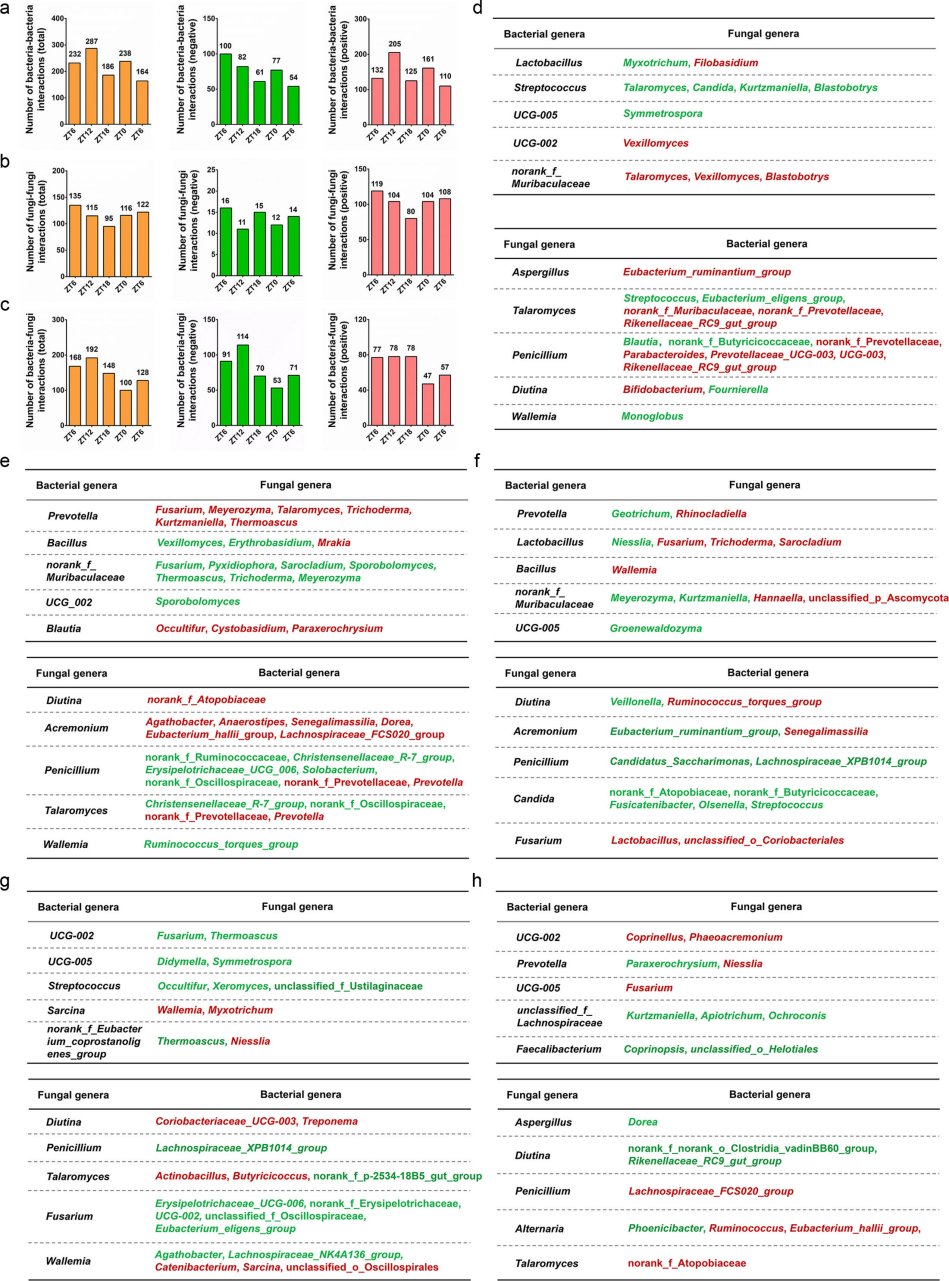
The rhythmic correlations of bacterial and fungal genera during a diurnal cycle. (a) The number of total, negative, and positive bacteria–bacteria interactions during a diurnal cycle. (b) The number of total, negative, and positive fungi–fungi interactions during a diurnal cycle. (c) The number of total, negative, and positive bacteria–fungi interactions during a diurnal cycle. The correlations between the top 5 bacterial or fungal genera with fungal or bacterial genera are analyzed at ZT6 (d), ZT12 (e), ZT18 (f), ZT0 (g), and ZT6 (h), respectively. The red genus represents positive correlations, while the green genus represents negative correlations.

By calculating the number of total, negative, and positive interactions between gut microbes, we found that the most positive bacteria–bacteria interactions (205) were observed at the B (ZT12) time point, while the negative bacteria–bacteria interactions (100) were abundant at the A (ZT6) time point (Fig. 5a). For the fungi–fungi correlation analysis, the most positive (119) and negative (16) interactions were observed at the A (ZT6) time point (Fig. 5b). For the bacteria–fungi correlation analysis, most of the positive interactions (78) occurred at the B (ZT12) and C (ZT18) time points, while the negative interactions (114) were abundant at the B (ZT12) time point (Fig. 5c). Of note, the fungal or bacterial genera that positively or negatively correlated with the top 5 bacterial or fungal genera were analyzed at the five time points, respectively (Fig. 5d through h).

## DISCUSSION

The rhythmic oscillation of gut microbiota exerts a significant influence on the normal physiology of the host and is greatly important for their health. However, most of the studies are mainly focused on the bacterial microbes, ignoring other components of gut microbes, such as the fungal microbes (mycobiota). Besides, the rhythmic correlations between the bacterial and fungal microbes were scarcely reported. In this study, we provided a comprehensive understanding of the rhythmic oscillations of bacterial and fungal communities in male cynomolgus monkeys and investigated their correlations during a diurnal cycle.

By comparing the rhythmic oscillation pattern of bacterial microbes between the mice, cynomolgus monkeys, and human beings, we found that both the bacterial richness and diversity exhibited obvious oscillations in mice (39) and human beings (40). During a 24-h period, the species richness and Shannon effective number of mice decreased from 0 to 10 h and then increased from 12 to 24 h. In human beings, these two parameters increased from 0 to 12 h and decreased from 16 to 24 h. However, both the Ace and Shannon indexes showed no obvious oscillations in cynomolgus monkeys (Fig. 1b).

As for the dominated bacterial taxa, we found that the relative abundance of Firmicutes exceeded Bacteroidota in cynomolgus monkeys (Fig. 1e) and human beings (40), while Bacteroidota represented the most abundant phylum in mice (39). Furthermore, we checked the rhythmic oscillation patterns of Firmicutes and Bacteroidota and found that the relative abundance of these two phyla exhibited obvious rhythmic oscillations in mice (39), cynomolgus monkeys (Fig. 1e), and human beings (40). In mice, the relative abundance of Firmicutes decreased from 0 to 12 h and then increased from 12 to 24 h, whereas the relative abundance of Bacteroidota showed the opposite trend (39). In human beings, the relative abundance of Firmicutes increased from 0 to 14 h and decreased from 14 to 24 h, while the relative abundance of Bacteroidota showed the opposite trend (40). In cynomolgus monkeys, our study indicated that the relative abundance of Firmicutes decreased from ZT6 (13:00) to ZT12 (19:00) and then increased from ZT12 (19:00) to ZT6 (13:00), whereas the relative abundance of Bacteroidota showed the opposite trend (Fig. 1e). As for the bacterial genera that oscillated rhythmically, *S24-7* spp. and *Lachnospiraceae* spp. were abundant in mice (13), while *Prevotella* and norank_f_Eubacterium_coprostanoligenes_group were enriched in male cynomolgus monkeys (Fig. 1f).

The fungal microbes represented a very small portion of gut microbiota. It had been reported that none of the highly abundant fungal genera (*Aspergillus*, *Talaromyces*, *Wallemia*, *Penicillium*, *Fusarium*, *Rhodotorula*, and *Sterigmatomyces*) exhibited significant rhythmic oscillations in mice (41). Differently, our results revealed that the relative abundance of *Aspergillus* and *Talaromyces* varied at ZT18 and exhibited slight oscillation patterns in male cynomolgus monkeys (Fig. 3h). These interspecies bacterial and fungal differences may be affected by the genetic and dietary factors of the host, which need to be verified in our future work.

The ecological interactions between microbes were widespread in almost all the environments (42, 43). Among these ecological systems, the gut microbiota represented a complex microbial community within the gastrointestinal (GI) tract of the host and played key roles in dietary nutrition and host physiology (44). Among the gut microbes, the bacterial and fungal microbes were two important microbial communities closely related to the health of the host (26, 32). Accumulating evidence indicated that the bacterial and fungal microbes could interact with each other to affect the growth, nutrition, reproduction, and pathogenicity of the host (45, 46). As for NHPs, although the site-specific correlations between the bacterial and fungal microbes were studied along the GI tract (19), the rhythmic interactions between these two microbial taxa were not reported. In this work, we analyzed the correlations between the bacterial and fungal microbes during a diurnal cycle and found that the bacterial and fungal microbes interacted with each other closely, with the most bacteria–fungi interactions occurring at ZT12 (Fig. 5c; Fig. S6). At this time point, *Prevotella*, the most abundant bacterial genus whose relative abundance oscillated rhythmically, positively interacted with the six fungal genera (*Fusarium*, *Meyerozyma*, *Talaromyces*, *Trichoderma*, *Kurtzmaniella*, and *Thermoascus*) (Fig. 5e). As reported, *Prevotella* microbes were highly abundant in various body sites of human beings, where they are key players in the balance between health and disease (47). Therefore, it needs to be further clarified how the normal physiological activities of the host are affected by the rhythmic interactions between the intestinal *Prevotella* microbes and fungal microbes.

### Limitations of this study

The limitation of our study is that we mainly focus on the male cynomolgus monkeys. Since the gut microbes underwent sex-dependent rhythmic alterations in rodents (13, 14), our results might not be applicable to the female cynomolgus monkeys, and further investigation is needed.

### Conclusion

Our study uncovers the diurnal oscillation patterns of bacterial and fungal microbes in male cynomolgus monkeys and investigates their correlations during a diurnal cycle. Compared with the fungal microbes, the bacterial microbes exhibited obvious rhythmic oscillations, especially for the genera norank_f_Eubacterium_coprostanoligenes_group and *Prevotella* whose relative abundance varied significantly at ZT12 (19:00). Consistent with this result, the gut bacterial functions were also oscillated significantly and closely correlated with the abovementioned two bacterial genera. By characterizing the bacterial and fungal interactions during a 24-h diurnal cycle, we found that the bacterial and fungal microbes closely interacted with each other, and the most bacteria–fungi interactions occurred at ZT12 (19:00).

## MATERIALS AND METHODS

### Animals

In this work, the eight captive male cynomolgus monkeys (*Macaca fascicularis*), with ages ranging from 2 to 3 years (the lifespan for cynomolgus monkeys was about 20–30 years), were housed at the Songjiang Non-human Primate Facility of Institute of Neuroscience. These monkeys were housed in an air-conditioned environment with controlled temperature (22°C ± 1°C), humidity (50% ± 5% RH), 12-h light/12-h dark cycle (lights-on time 07:00–19:00), and continuous access to municipal water. As for the diet, these monkeys were fed rhythmically with commercial monkey diet (Anmufei, Suzhou) twice daily (200 g per monkey at 8:00 and 15:00) and fruits and vegetables once daily (100 g per monkey at 10:00) to provide essential nutrition and vitamins.

Before the experiment, all the monkeys were housed singly for 2 months and had no antibiotic exposure. Then, the fecal samples of the single-caged monkeys were collected rhythmically at ZT6 (13:00), ZT12 (19:00), ZT18 (1:00), ZT0 (7:00), and ZT6 (13:00) during a 24-h diurnal cycle. The size of single cages used in this study was 0.7 m × 0.9 m × 1 m.

### Fecal sample collection

The fresh fecal samples of cynomolgus monkeys were collected by experienced working staffs at ZT6 (13:00), ZT12 (19:00), ZT18 (1:00), ZT0 (7:00), and ZT6 (13:00) during a 24-h diurnal cycle. The time used to collect the fecal samples at each of the time points was about 1 h. The collected samples were frozen immediately with liquid nitrogen in sterile tubes and stored at −80°C until use.

### Gut microbiome sequencing analysis

The total genomic DNA samples were extracted by using the Omega Soil DNA Kit (M5635-02) (Omega Bio-Tek, Norcross, GA, USA). The quality and concentration of DNA were determined by 1.0% agarose gel electrophoresis and a NanoDrop ND-2000 spectrophotometer (Thermo Scientific Inc., USA) and kept at −80°C prior to further use. For 16S rRNA amplicon sequencing, the hypervariable V3-V4 region of 16S rRNA was amplified using the primer pairs 338F (5′-ACTCCTACGGGAGGCAGCA-3′) and 806R (5′-GGACTACHVGGGTWTCTAAT-3′) by an ABI GeneAmp 9700 PCR thermocycler (ABI, California, USA). For ITS amplicon sequencing, the fungal ITS1-2 regions were amplified by PCR using the following primer pairs ITS1F (5′-CTTGGTCATTTAGAGGAAGTAA-3′) and ITS2R (5′-GCTGCGTTCTTCATCGATGC-3′). The PCR mixture included 4 µL 5 × Fast Pfu buffer, 2 µL 2.5 mM dNTPs, 0.8 µL each primer (5 µM), 0.4 µL Fast Pfu polymerase, 10 ng of template DNA, and ddH_2_O to a final volume of 20 µL. The PCR amplification cycling conditions were as follows: initial denaturation at 95°C for 3 min, followed by 27 cycles of denaturation at 95°C for 30 s, annealing at 55°C for 30 s and extension at 72°C for 45 s, and single extension at 72°C for 10 min, and end at 4°C. Then, the PCR products were purified using the AxyPrep DNA Gel Extraction Kit (Axygen Biosciences, Union City, CA, USA) and quantified using Quantus Fluorometer (Promega, USA). Equal amounts of amplicons were used for paired-end sequencing using the Illumina MiSeq PE300 platform (Illumina, San Diego, USA) according to the standard protocols by Majorbio Bio-Pharm Technology Co. Ltd. (Shanghai, China).

### Data processing

Raw FASTQ files were de-multiplexed using an in-house Perl script, quality-filtered by fastp version 0.19.6 (48), and merged by FLASH version 1.2.11 (49) with the following criteria: (i) the 300-bp reads were truncated at any site receiving an average quality score of <20 over a 50-bp sliding window, the truncated reads shorter than 50 bp were discarded, and reads containing ambiguous characters were also discarded; (ii) only overlapping sequences longer than 10 bp were assembled according to their overlapped sequence. The maximum mismatch ratio of the overlap region is 0.2. Reads that could not be assembled were discarded. (iii) Samples were distinguished according to the barcode and primers, and the sequence direction was adjusted, with exact barcode matching and two nucleotide mismatch in primer matching. The high-quality sequences were de-noised using DADA2 and viewed as ASVs. The taxonomic assignment of ASVs was performed using the Naive Bayes consensus taxonomy classifier implemented in Qiime2 and the SILVA 16S rRNA database (v138) and ITS database (UNITE 8.0). The bacterial and fungal function was predicted by PICRUSt2 (50) and FUNGuild based on ASV representative sequences.

### Bioinformatics and statistical analysis

Bioinformatic analysis of the gut microbiota was carried out using the Majorbio Cloud platform (https://cloud.majorbio.com). The rarefaction curves and α-diversity indices including ACE, Shannon index, and Good’s coverage were calculated with Mothur v1.30.2 (51). The similarity among the microbial communities in different samples was determined by PCoA based on weighted UniFrac distances using the Vegan v2.4.3 package. The PERMANOVA test was used to assess the percentage of variation explained by the treatment along with its statistical significance using the Vegan v2.4.3 package. A Venn diagram was generated using R package “VennDiagram.” The LEfSe (52) (http://huttenhower.sph.harvard.edu/LEfSe) was performed to identify the significantly abundant taxa (phylum to species) of bacteria and fungi among the different groups.

### Correlation network analysis

Gephi software (version 0.9.4) was used to perform the correlation network. Spearman correlation analysis was used to uncover the bacterial–fungal correlations at ZT6, ZT12, ZT18, ZT0, and ZT6. A connection is indicated for Spearman’s correlation with a coefficient >0.6 (positive correlation) or <−0.6 (negative correlation) and a significant (*P* < 0.05) correlation. In the correlation network, bacteria are labeled as a yellow node; fungi are labeled as a green node. The size of each node is proportional to the relative abundance. The red lines represent positive correlations between the nodes, and the green lines represent negative correlations, with the line width indicating the correlation magnitude.

### Statistical analysis

The statistical analysis data were presented as the mean ± SEM. The statistical significance between two time points was analyzed using the Student *t*-test (unpaired, two-tailed). All statistical analyses were calculated by using GraphPad (version 7.0).

## Data Availability

The 16S rRNA and ITS amplicon sequencing data used in this study have been deposited in the Sequence Read Archive of NCBI (https://submit.ncbi.nlm.nih.gov/) with accession nos. SRP485888 (PRJNA1068982) and SRP485542 (PRJNA1068286).

## SUPPLEMENTAL MATERIAL

Supplemental figures spectrum.00722-24-s0001.docx Figures S1 to S6.

Table S1 spectrum.00722-24-s0002.xlsx Bacteria-fungi correlations at ZT6 (A time point).

Table S2 spectrum.00722-24-s0003.xlsx Bacteria-fungi correlations at ZT12 (B time point).

Table S3 spectrum.00722-24-s0004.xlsx Bacteria-fungi correlations at ZT18 (C time point).

Table S4 spectrum.00722-24-s0005.xlsx Bacteria-fungi correlations at ZT0 (D time point).

Table S5 spectrum.00722-24-s0006.xlsx Bacteria-fungi correlations at ZT6 (E time point).

ASM does not own the copyrights to Supplemental Material that may be linked to, or accessed through, an article. The authors have granted ASM a non-exclusive, world-wide license to publish the Supplemental Material files. Please contact the corresponding author directly for reuse.
