# Supplementary material for: Characterizing the rhythmic oscillations of gut bacterial and fungal communities and their rhythmic interactions in male cynomolgus monkeys
Source: Microbiol Spectr. 2024 Sep 25;12(11):e00722-24. doi: 10.1128/spectrum.00722-24 (PMC11537094; doi:10.1128/spectrum.00722-24)
Supplement: Supplemental figures — Figures S1 to S6. [file spectrum.00722-24-s0001.docx]

**Supplementary information for:**

**Characterizing the rhythmic oscillations of gut bacterial and fungal communities** **and their rhythmic interactions in male** **cynomolgus monkeys**

Yunpeng Yang^1,2,3,4,5,*^, Meiling Yu^1,2,5^, Yong Lu^3^, Changshan Gao^3^, Ruxue Sun^1,2^, Wanying Zhang^1,2^, Yanhong Nie^3,4^, Xinyan Bian^3^, Zongping Liu^1,2,*^, Qiang Sun^3,4,*^

^1^Jiangsu Co-innovation Center for Prevention and Control of Important Animal Infectious Diseases and Zoonoses, College of Veterinary Medicine, Yangzhou University, Yangzhou, 225009, China

^2^Institute of Comparative Medicine, Yangzhou University, Yangzhou, 225009, China

^3^Institute of Neuroscience, CAS Key Laboratory of Primate Neurobiology, State Key Laboratory of Neuroscience, CAS Center for Excellence in Brain Science and Intelligence Technology, Chinese Academy of Sciences, Shanghai, 200031, China

^4^Shanghai Center for Brain Science and Brain-Inspired Technology, Shanghai, 201602, China

^5^Yunpeng Yang and Meiling Yu contributed equally to this article

^*^Address correspondence to Zongping Liu, liuzongping@yzu.edu.cn, Qiang Sun, [qsun@ion.ac.cn](mailto:qsun@ion.ac.cn), or Yunpeng Yang, [ypyang@yzu.edu.cn](mailto:ypyang@yzu.edu.cn,)


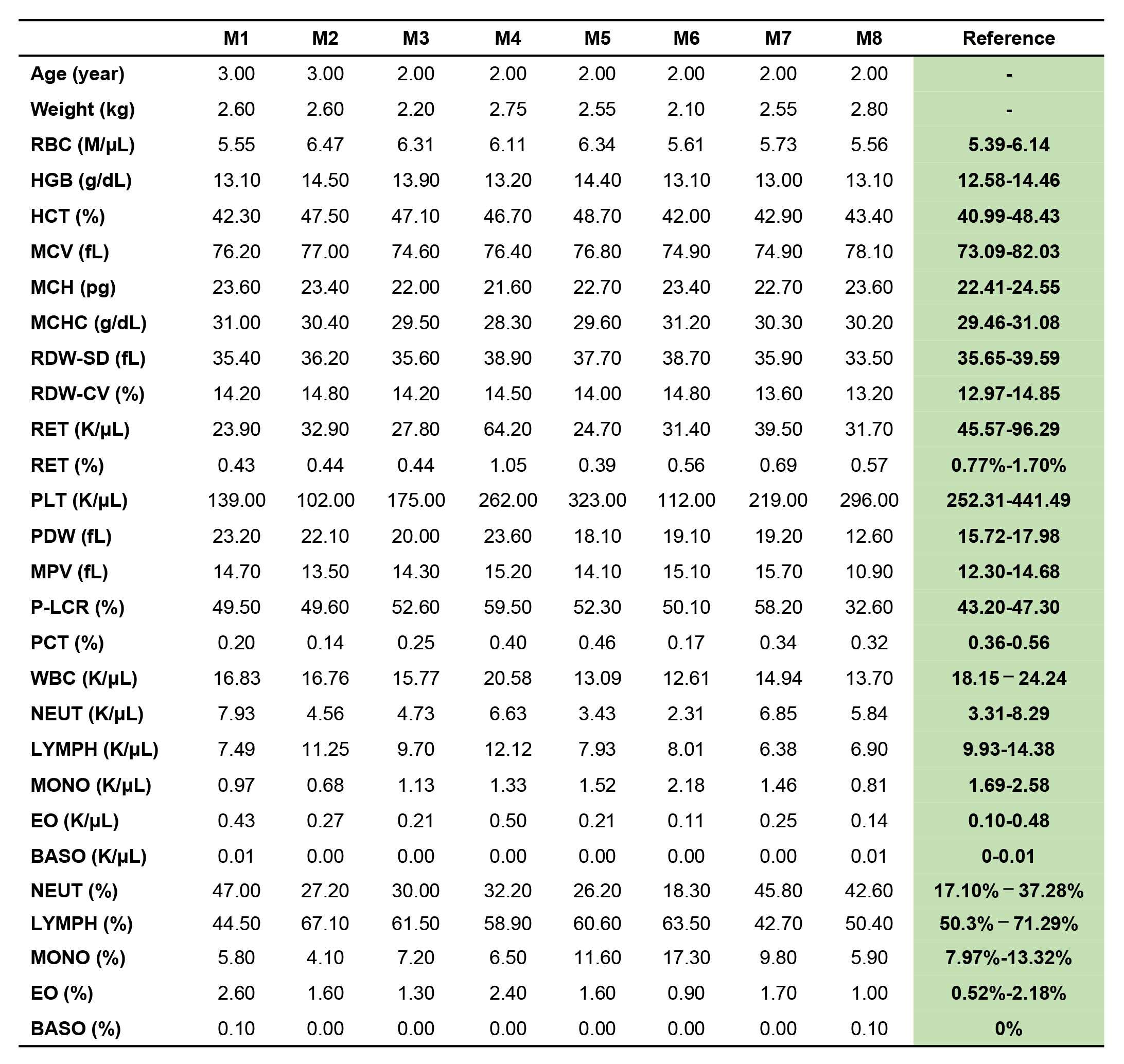


**FIG S1** The routine blood indexes of eight male cynomolgus monkeys used in this study. The eight monkeys are labeled as M1 to M8.


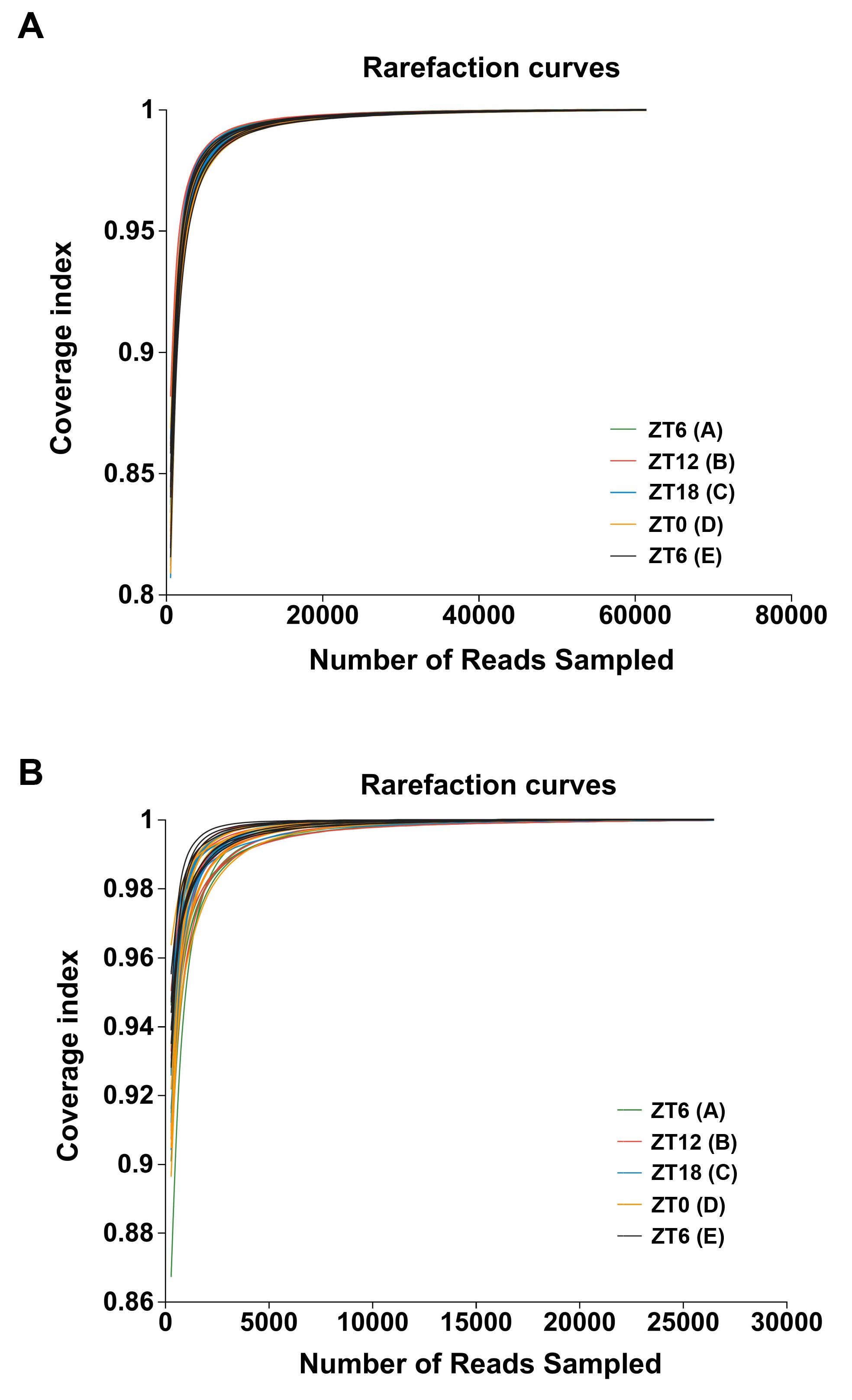


**FIG S2** The average Good’s coverage of each sample at ZT6, ZT12, ZT18, ZT0, and ZT6 in the 16S rRNA (A) and ITS (B) amplicon sequencing data.


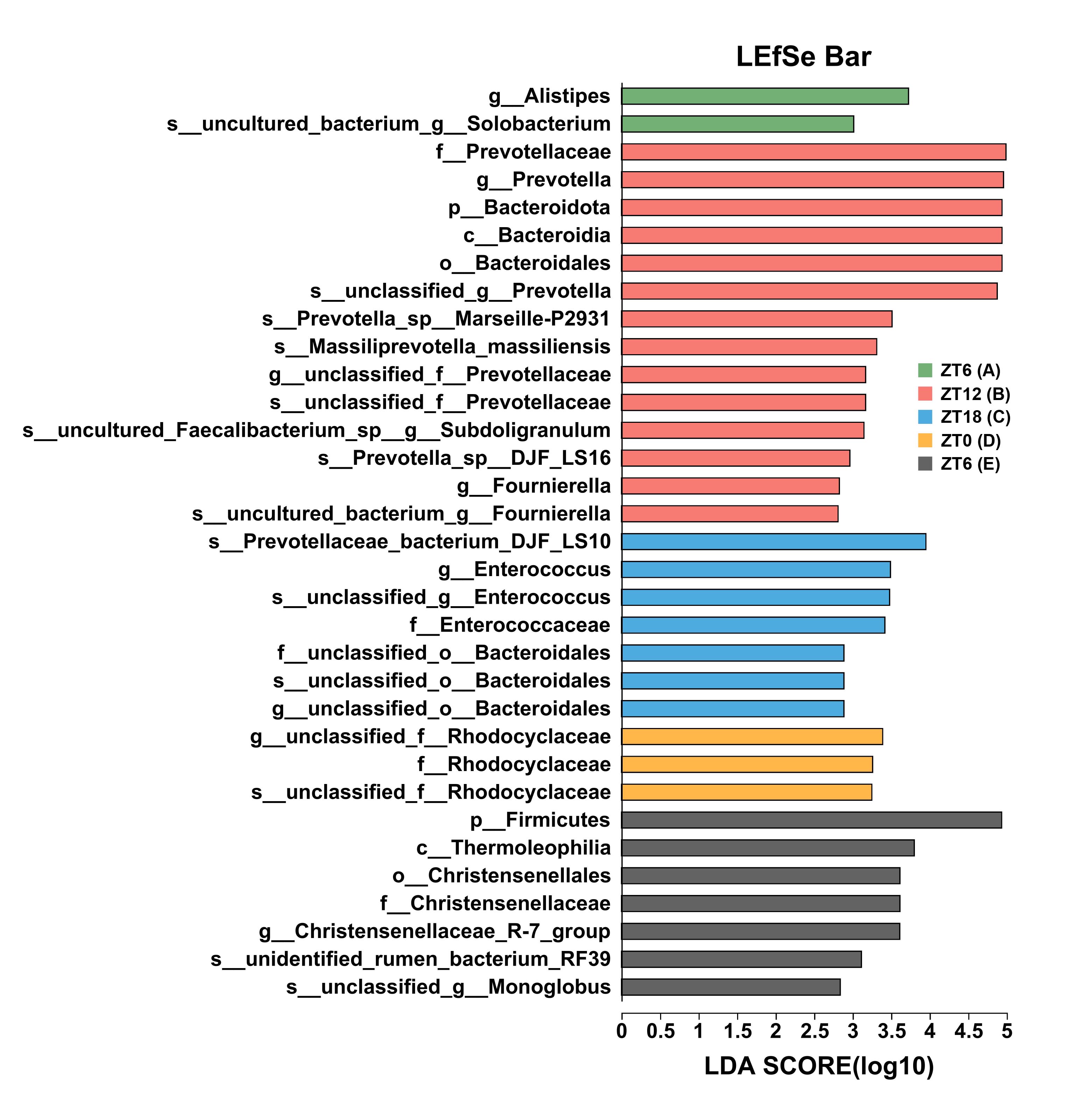


**FIG S3** LEfSe analysis to characterize the taxonomic differences of bacterial microbes between ZT6 (A), ZT12 (B), ZT18 (C), ZT0 (D), and ZT6 (E). LDA score cut-off was set as 2.0 (P<0.05).


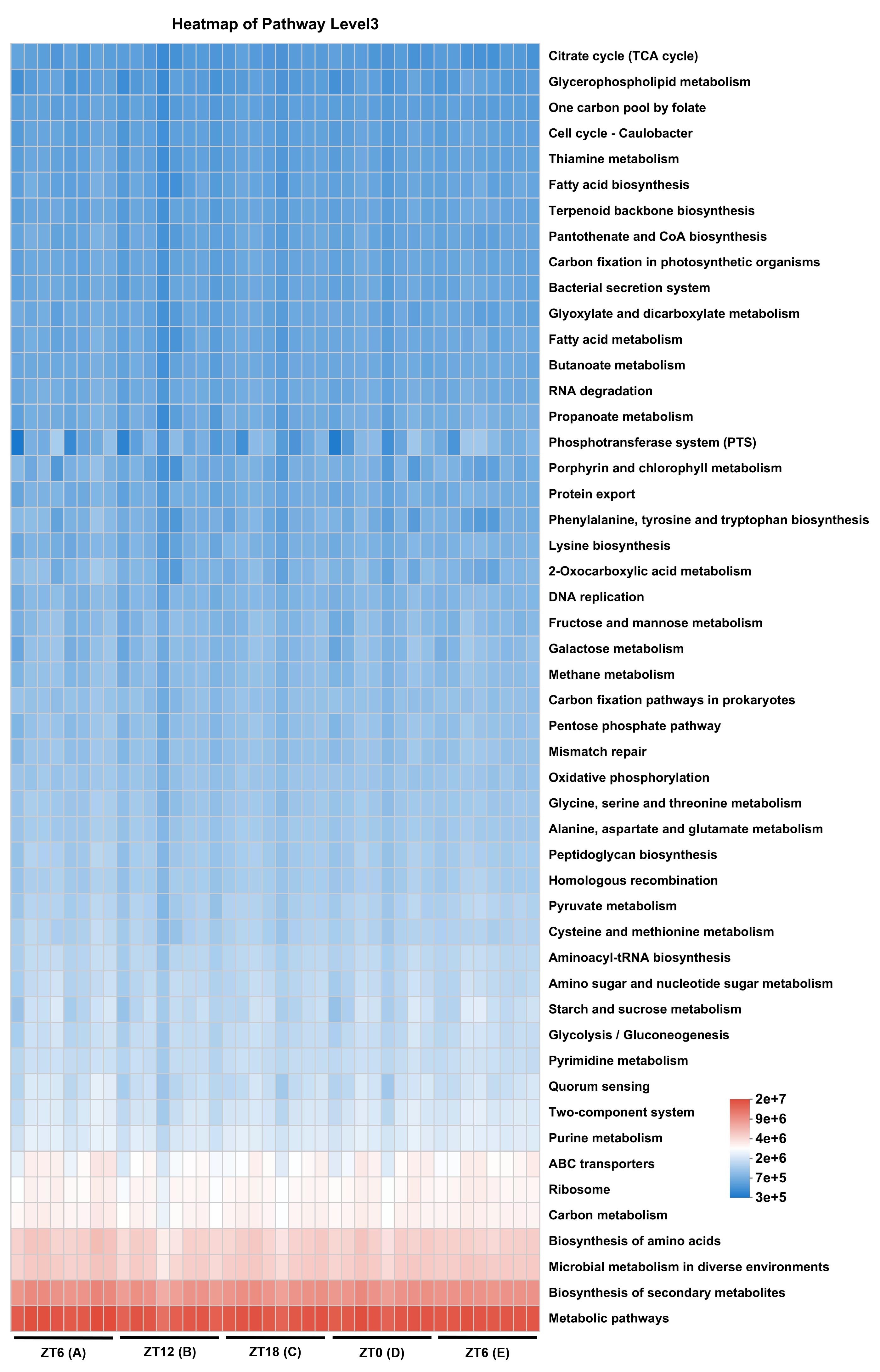


**FIG S4** Heatmap illustrating PICRUSt2-predicted gut bacterial functions at ZT6 (A), ZT12 (B), ZT18 (C), ZT0 (D), and ZT6 (E) in 16S rRNA amplicon sequencing related to KEGG pathways at level 3


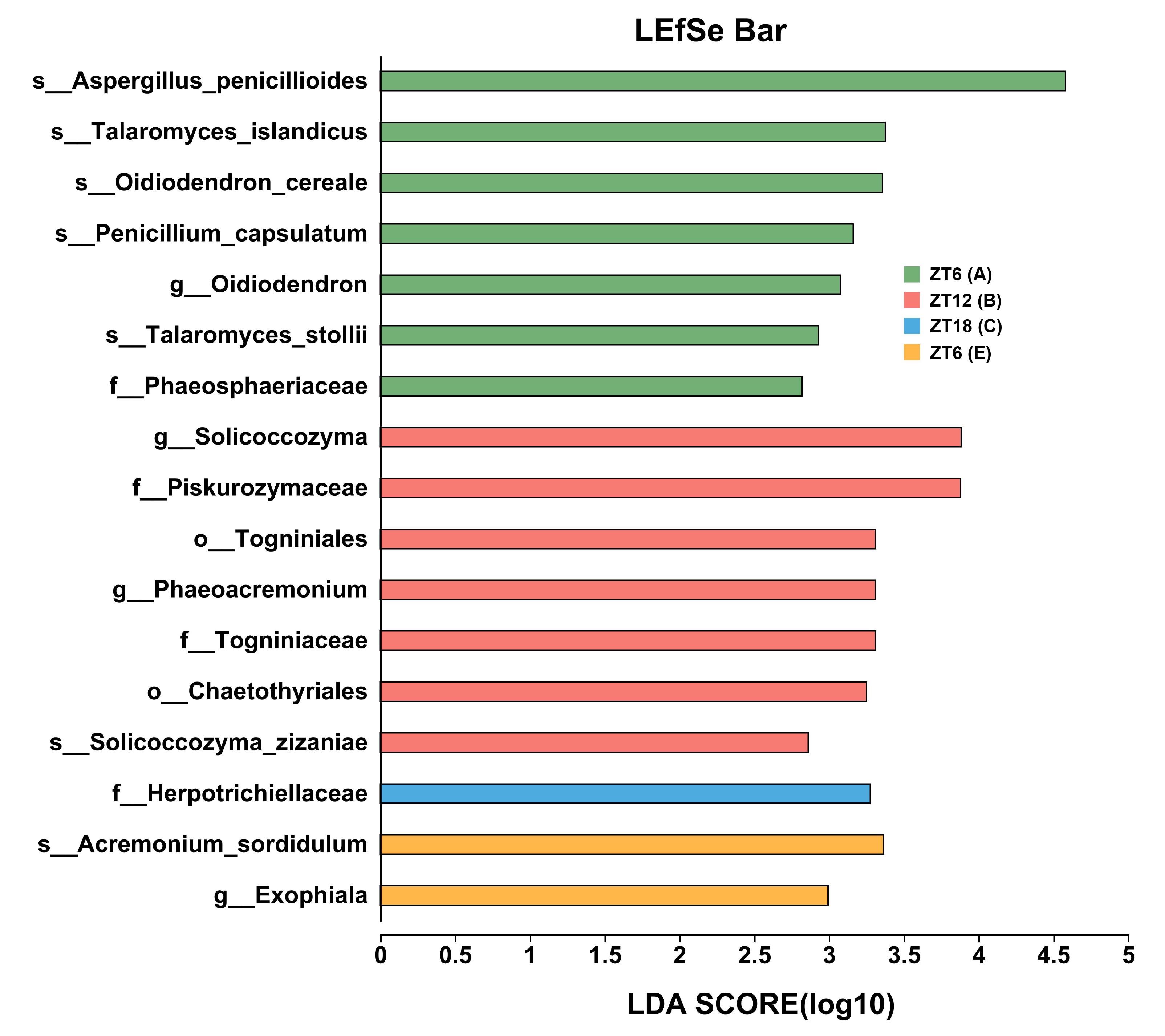


**FIG S5** LEfSe analysis to characterize the taxonomic differences of fungal microbes between ZT6 (A), ZT12 (B), ZT18 (C), and ZT6 (E). LDA score cut-off was set as 1.0 (P<0.05).





**FIG S6** Correlation networks of bacterial and fungal genera at five time points: A (ZT6), B (ZT12), C (ZT18), D (ZT0), and E (ZT6). The correlation network is performed by using Gephi software (version 0.9.4). A connection is indicated for Spearman’s correlation with a coefficient >0.6 (positive correlation) or <−0.6 (negative correlation) and a significant (P < 0.05) correlation. Bacteria are labeled as a yellow node; fungi are labeled as a green node. The size of each node is proportional to the relative abundance. The red lines represent positive correlations between the nodes, and the green lines represent negative correlations, with the line width indicating the correlation magnitude.
